# Ardulake temperature profiler: An open-source, low-cost, automated monitoring system to unravel the mixing behavior of lakes

**DOI:** 10.1016/j.ohx.2024.e00606

**Published:** 2024-11-14

**Authors:** Guillermo Goyenola, Javier García-Climent

**Affiliations:** Laboratorio de Ciencia de Cuencas y Limnología del Antropoceno, Departamento de Ecología y Evolución, Centro Universitario Regional del Este, Universidad de la República, Maldonado, Uruguay

**Keywords:** Temperature monitoring system, Lake mixing, Thermal stratification, Monitoring buoy, Limnology

## Abstract

Understanding the thermal classification of lakes based on mixing regimes is fundamental in limnology. Although this classification has traditionally been considered well-established, recent studies highlight variations in the mixing behaviors of ponds and shallow lakes. This paper introduces the Ardulake temperature profiler, an innovative, simple, and autonomous high-frequency temperature monitoring system designed for shallow to moderately deep lakes (3.5 to 10 m). Utilizing Arduino technology and GPRS telemetry, the system is cost-effective, with electronic components and sensors costing approximately USD 250 and buoy construction and deployment around USD 1000. The Ardulake enables real-time environmental temperature monitoring and data storage on an online platform for subsequent analysis and visualization. The collected data supports ecosystem research and the numerical modeling of thermal behavior in lakes. Key strengths of the system include low production and maintenance costs, replicability, and customization capabilities. Challenges, such as interference from animal activity, are addressed with recommended preventive measures tailored to specific fauna. Overall, the Ardulake temperature profiler offers a practical tool for advancing limnological research, with potential for modification to various environmental monitoring objectives.

## Graphical abstract

**Figure:**
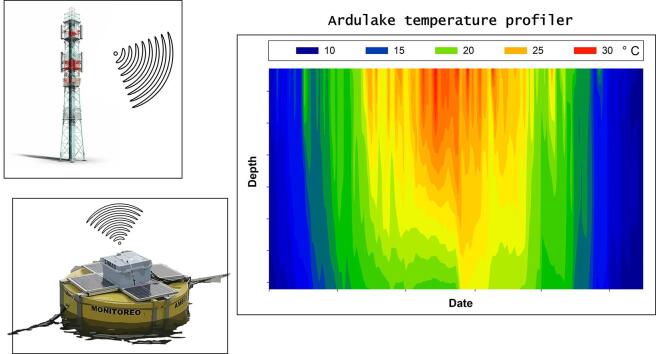

## 1 Specifications table

**Table:** 

Hardware name	Ardulake temperature profiler
Subject area	Aquatic sciences
Hardware type	Field measurements
Closest commercial analog	NexSens T-Node FR Thermistor String, X3 Environmental Data Logger, CB-450 Data Buoy, WQData LIVE Web Datacenter
Open source license	CC BY-SA 4.0 DEED
Cost of hardware	$1,250
Source file repository	https://doi.org/10.17632/wnxv7wbfsx.5

## 2 Hardware in context

Over the past fifteen years, significant advancements have been made in the development of environmental sensors, ranging from low-cost, robust data loggers for monitoring stream temperature and water quality [1] to more sophisticated, real-time, autonomous systems for ecosystem monitoring [2]. For example, low-cost prototyping systems for sensor networks [3] have facilitated widespread deployment in various environmental settings. These innovations have bridged the gap between affordability and precision, enabling both large-scale and fine-grained environmental monitoring across diverse ecosystems. However, despite these technological advances, there has been a noticeable gap in the development of specialized equipment for assessing thermal stratification and mixing regimes in lakes, which are critical for understanding lake dynamics and their ecological implications.

Factors such as climate, meteorology, altitude, and morphology (and their interactions) are the main aspects determining lake cycling, productivity, and the effects of nutrient pollution [4], [5], [6], [7]. In lakes, major heat exchanges occur at the surface, and if the wind mixing is weak, vertical temperature gradients can determine the differentiation of the density of water masses [8]. Buoyancy differences resulting from these gradients are enough to determine the vertical distribution of substances (such as nutrients, dissolved oxygen, and H^+^), particles, and organisms, conditioning ecosystem functioning and water quality.

The thermal classification of lakes according to their mixing regime is a piece of classical limnology that accumulated decades of research [9], [10]. Lakes below 5 m are traditionally classified as shallow and historically categorized as continuously mixing systems, leading to intense recycling of substances and strong biological productivity [6]. On the other hand, deeper lakes have a much lower frequency of vertical mixing [9], [10]. The topic seemed to be scientifically settled, but new knowledge is emerging about differences in mixing regimes still for ponds and shallow lakes [6], [11], [12].

Shallow or slightly deep lakes (3.5 to 10 m depth) can behave differently depending on the combination of their conditions and external influences. Therefore, it is crucial to enhance our understanding of their temporal dynamics and how they influence their functioning and interaction with society [13].

Automated monitoring of lakes allows scientists to investigate the thermal stratification and mixing regimes of lakes with spatial and temporal detail. Using open-source technologies and data telemetry offers several advantages over traditional manual monitoring methods [14], [15]. One significant benefit is the ability to provide continuous and real-time monitoring of environmental conditions, ensuring uninterrupted data collection and eliminating the risk of data loss due to human factors such as worker absence or influence [16], [17]. Automated monitoring is also a reliable solution to the challenges faced during pandemics. The COVID-19 pandemic generated significant difficulties in maintaining environmental monitoring programs due to mobility restrictions and social distancing measures imposed in some countries. These restrictions limited the ability of scientists and technicians to access field sites, leading to disruptions in data collection and gaps in long-term environmental records. By ensuring consistent and high-quality data collection even during restricted access, automated monitoring helps to maintain the continuity and reliability of environmental observations. Another advantage of an automated monitoring system is that it can be programmed to collect data concomitantly at different sites (as specific depths within a lake), providing a more comprehensive view of the ecosystem’s properties.

In recent years, the number of options to monitor aquatic systems has experienced significant growth with a long list of companies specialized in the sector (e.g. Yellow Springs Instruments, In-Situ Inc.). However, the available options are expensive and set technical restraints that may hinder certain research objectives. Developing low-cost monitoring equipment can help overcome these challenges, providing researchers with affordable and tailored solutions to meet their research needs. Moreover, customized solutions can offer technical advantages over commercial equipment by allowing users to modify and adapt their capabilities. This enhances the accuracy and reliability of data collection, enabling users to leverage the technology and reduce their dependence.

Open-source technologies offer great potential for customization for monitoring systems to meet specific needs while facilitating sharing and collaboration among researchers. Telemetry of data enables remote access to collected data, allowing researchers to analyze it from anywhere in the world and reducing the need for costly and time-consuming site visits. Using automatic monitoring systems, employing open-source technologies and data telemetry provides a more comprehensive, accurate, and efficient approach to monitoring lake conditions. This allows researchers to understand lake dynamics better, leading to more informed management decisions.

Arduino is an open-source electronics platform based on easy-to-use hardware and software (https://www.arduino.cc/). It is intended for anyone doing interactive projects. This technology allows us to investigate the environment by receiving inputs from many sensors. There are several publications of Arduino-based developments, including wave height estimation [18], movement of organisms [19], and physicochemical variation [20], [21], [22], demonstrating the potential of this monitoring strategy of environmental monitoring.

The present article presents the technological development of an original, simple, and autonomous high-frequency temperature monitoring system at various depths applied to research the thermal behavior of not-so-shallow lakes. The proposed monitoring system, the Ardulake temperature profiler, was based on Arduino technology and utilized telemetry via GPRS. We describe the development, building, installation, and evaluation based on performance. Advice for developing projects with similar characteristics is also provided and the possibility of modification to suit a diverse range of objectives is proposed.

## 3 Hardware description

The monitoring system was developed as a floating platform that supports solar-powered equipment that periodically senses and transmits data by GPRS to a data center. Electronics are based on Arduino Nano R3 microcontroller, a small, low-cost board based on the ATmega328P chip. The string of sensors was built with Dallas DS18B20 digital temperature sensors. This equipment sends this data through a SIM800L GSM module to be stored on an Internet of Things (IoT) platform.

**Bullet points**

• The Ardulake temperature profiler monitoring system is useful for real-time high-frequency monitoring of the environmental temperature of shallow or slightly deep lakes (3.5 to 10 m), as well as storing the data on an online platform for further analysis or visualization.
• The main strengths of the Ardulake temperature profiler system are simplicity, low production and maintenance costs, replicability, and customization possibilities.
• The generated data is adequate to support ecosystem research and numerically modeling the thermal behavior of lakes.
• Based on our experience, the major problems using the Ardulake temperature profiler monitoring system arose because of the animal’s activity. A preventive approach is recommended in this regard, which must be adapted depending on the characteristics of the fauna present.

## 4 Design files summary

**Table:** 

Design filename	File type	Open-source license	Location of the file in [23]
01- ArduLake- schematics	.pdf	CC BY 4.0	Folder “01-Schematics”
02- ArduLake- with solar panels	.fzz (https://fritzing.org/)	CC BY 4.0	
03- ArduLake- without solar panels		CC BY 4.0	
Buoy and deployment design	.pdf	CC BY 4.0	
Ardulake temperature profiler − Arduino nano programming code	.docx	CC BY 4.0	02- Source Code
Updated_Ardulakecode	.ino (Code; Arduino IDE format)	CC BY 4.0	
Suppl 1 Step-by-step guide to customize the Arduino nano code	.pdf	CC BY 4.0	03- Step-by-step guides
Suppl 2 Step-by-step guide to work with the Arduino nano code		CC BY 4.0	
Suppl 3 Step-by-step guide to adding additional temperature sensors		CC BY 4.0	
01-temp&density_plots-Goyenola&Garcia2024	.R (Script − RStudio desktop)	CC BY 4.0	04- data and R-Scripts
01-temperature-density-stratification	.xlsx (data Worksheet)	CC BY 4.0	
02-stratification_probability		CC BY 4.0	
02-vertical temperature gradients-Goyenola&Garcia2024	.R (Script − RStudio desktop)	CC BY 4.0	
index_3d_english	.html (interactive graph)	CC BY 4.0	
information	.txt	CC BY 4.0	
Image archive (from first prototype in 2019).pdf	.pdf	CC BY 4.0	05-Image archive (from first prototype in 2019)

## 5 Bill of materials summary

**Table:** 

Designator	Component	Number	Cost per unit (USD)	Total cost (USD)	Source of materials
**Electronics**	Arduino Nano (Rev3.0)	1	13	13	www.amazon.com
	SIM800L GSM/GPRS module	1	9	10	
	LM2596 DC-DC Buck converter step-down module	1	7.5	10	
	Relay Module KY-019	1	8	9	
	Toggle Switch	1	7	7	
	1MΩ, 100kΩ, 4.7 Ω Resistors, tolerance ± 5 %	Kit	11	11	
**Sensors**	DS18B20 Wire Temperature Sensor Probes	6	3	18	
**Power**	10 Watts 12 Volt Solar Panel	4	30	120	
	Solar Charge Controller	1	12	12	
	12 V/7Ah battery	1	40	40	
**Buoy parts and accessories**	300 L water tank	1	150	150	plumbing supplies store
	Polypropylene thermofusion pipes	1 m	5	5	
	Polyurethane foam spry	At least 10	15 by foam gun canister	150	hardware store
	Paint for plastic surfaces(Rust-Oleum Ultra Cover 2x Sun-yellow, glow (430 mL)	5 cans	15	75	
	Silicone sealant	100 mL container	5	5	
	Stainless steel straps and buckles	7 m and 2 buckles	4 + 1	30	
	Rectangular profile aluminum tubes and aluminum angles (50 x 25 mm)	5 x 3 m	15	75	aluminum carpentry supplies store
	Pop rivets and stainless-steel screws	140 + 70		20	electrical shop (Maudy S.A., Atlántida)
	Weather-resistant plastic straps	As many as necessary	20	20	
	Rugged plastic waterproof box with cable glands (we use polypropylene Roker box, 315 x 330 x 180 mm)	1	60	60	electrical shop (Electro Palace, Montevideo)
	Cables and connectors	As many as necessary	15	15	
	Containers with silica gel	500 g	15	15	chemical wholesaler
	2 Nautical buoys (we used spheric Ф **=** 30 cm, made of PVC plastisol (UV resistant)	2	50	100	nautical store (Proamar & Todosailing)
	Nautical ropes (Ф 10 to 12 mm)	30 m (depending on depth)	3	90	
	Inox nautical chains (6 mm AIS316 DIN 766 or similar)	2 sections of 2 m	22	88	
	4 mm stainless steel rope-rubber coated and cable clamps	3.5 m	20	70	
	Concrete deadweights(we use reinforced concrete columns with internal rods; 120 x 10 x 10 cm)	2	8	16	construction materials store
	Spikes, plastic net or bird deterrent materials (depending on the behavior of specific fauna)	As many as necessary	20	20	mercadolibre.com.uy

The cost of working hours for the equipment development was not estimated.

## 6 Build instructions

### 6.1 Electronics

**Fig. 1:**
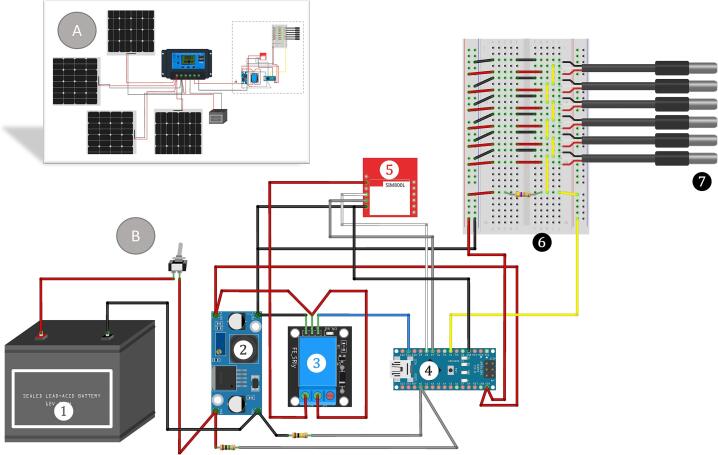
Monitoring assembly circuit of Ardulake temperature profiler. A: Complete diagram including solar panels and charge controller. B: Zoom of main components. (1) Battery; (2) LM2596-Converter; (3) relay; (4) Arduino Nano; (5) SIM800L; (6) breadboard (included only to show the connections clearly); (7) digital temperature sensors. All the connections were tin-soldered. The complete fritzing diagram can be found in the associated repository [23].

The Arduino Nano R3 microcontroller (Fig. 1) has 14 digital input/output pins and six analog inputs and operates on a 5 V DC power supply. It also includes a USB port for serial communication. The board can be programmed using Arduino Integrated Development Environment (IDE). The step-by-step guide to working with the Arduino Nano code can be found in the associated repository [23].

Six Dallas DS18B20 digital temperature sensors have been installed, one to measure air temperature and five at different depths. The performance of temperature sensors was tested placing the six sensors in a controlled environment at 20 °C, where each sensor recorded four measurements. An analysis of variance (ANOVA) discarded significant differences between the sensors (F_(5,18)_ = 0.188; p = 0.963). Accuracy was guaranteed using a one-sample *t*-test performed for each sensor. The comparison of the sensor readings to the expected value of 10.0° C does not produce significant differences (0.888 > t_(3)_ > 2.245; 0.113 > p > 0.437).

A resistive voltage divider connected to analog pin 0 allows voltage, which feeds the equipment, to be measured. To lengthen the 3 m sensor cables, they were soldered and insulated meticulously using heat shrink spaghetti and self-vulcanizing tape. The sensor cables were protected by placing them inside a flexible black hose weighed down and hanging from the buoy. The sensitive metallic extreme of each sensor was situated at a fixed distance from the surface. We chose 0.15, 0.6, 1.6, 2.6, and 3.6 m deep to place the sensors, but these depths can be modified depending on research objectives and the lake’s maximum depth. The installation method and placement of the air sensor required iterative adjustments involving multiple tests. All alternatives tested on the upper part of the buoy exhibited significant overheating due to direct sunlight exposure and were therefore discarded. It was concluded that the most suitable position for the sensor is to attach it to the side of the buoy, a few centimeters above the waterline. Given that the buoy is installed at 34° South, the southern face was chosen so the sensor would always remain in the buoy’s shade. It should not be overlooked that the final installation of the air temperature sensor may bias the readings on rainy and/or stormy days due to the wave action. The air temperature data recorded by the Instituto Nacional de Meteorología’s station at Carrasco International Airport, located 2.8 km from the lake, was used as a reference.

The Ardulake temperature profiler sends temperature and voltage data through a SIM800L GSM module with a 2G compatible Micro SIM card to be stored on an Internet of Things (IoT) platform. The code uses the OneWire.h and DallasTemperature.h libraries to communicate with the temperature sensor and the SoftwareSerial.h library to communicate with the SIM800L module through digital pins. To save battery consumption, the program activates a relay connected to pin 11 of Arduino to turn on the SIM800L, starts serial communication with it, waits for 20 s for the module to connect to the cellular network, and sends the temperature and voltage data to ThingSpeak (https://thingspeak.com/) using a Write API Key. Finally, it takes an hour before the data is measured and sent again. It also uses the MsTimer2.h library for functioning testing to generate periodic interruptions and the flash function to turn on and off the LED on pin 13 every second.

Four 10 W solar panels provide the power source. This oversized charging station compensates for the lack of efficiency in its orientation and reduces maintenance costs. A solar charge controller stores power in a 12/7Ah non-spillable sealed lead-acid battery, which feeds the monitoring equipment. To transform 12 V to 5 V, a step-down module, eBoot-LM2596-Converter-3-0-40 V-1-5-35 V, was used. To reduce unnecessary power consumption, a one-channel relay Module KY-019 was used to turn on the SIM800L only when it needed to send data. The device is powered on and off using the toggle switch. No additional steps are required for it to begin taking periodic measurements once it is turned on.

### 6.2 Code customization

The Arduino Nano programming code is available at in the associated repository [23] as a.ino file. The.ino file is a plain text file containing code written in a language based on C/C++ for use with the Arduino development environment. This file extension is specific to the Arduino IDE software (https://www.arduino.cc/en/software), which recognizes it as a sketch file, but at its core, it is just a text file with a different extension. You can open and edit.ino files with any text editor, such as Notepad on Windows, TextEdit on macOS, or any other code editor like Visual Studio Code or Sublime Text. However, it’s best to open.ino files using the Arduino IDE because it provides additional features such as syntax highlighting, code verification, and uploading directly to the Arduino board.

Before using the provided Arduino code, you must customize the transmission and receiving data information. We also provide in the associated repository [23] the code as a Microsoft Word file, where you can find yellow-marked text that must be substituted. If you use the Word file for the customization, you must delete the first references (all the text before the # sign) and save it as a.txt file. After that, you must change the file extension to.ino. Follow instructions below, or consult the step-by-step guide to code customization in Supplementary Material 1 available in the associated repository [23]. After customizing your code, you must open the Arduino IDE software and follow the step-by-step instructions included as Supplementary Material 2 [23].

*1st step on code customization: GPRS network configuration*

The user must buy a 2G compatible Micro SIM card and charge credit for SMS transmission. Momentarily insert the SIM into a mobile phone device and set it so that it is unnecessary to enter the personal identification number (PIN code) again. In an Android device, you must open Settings > Security and privacy > SIM card lock option > deactivate it. For security reasons, you must enter the PIN one last time before confirming it. Get the information about the Access Point Name (APN) specific to the company providing the service.

Essentially, the APN determines how your device connects to the internet through your mobile carrier’s network. This data must be provided by the company that provides the Micro SIM CARD. Some companies also provide usernames and passwords associated with an APN.

You must open the provided Arduino code in Microsoft Word format, find the line that starts with “Sim900Serial.println,” and substitute yellow-marked text with APN, username, and password without using spaces. It is important to double-check this configuration. If no username and password are needed, delete the correspondent painted text.

Example:

**Table:** 

Sim900Serial.println(“AT + CSTT=\”yourAPN\“,\”yourUsername\“,\”yourPassword\“”);command configures the APN
delay(1000);

*2nd step on code customization: Data reception using ThingSpeak*

We used ThingSpeak, an IoT analytics platform service that allows you to aggregate, visualize, and analyze live data streams in the cloud. ThingSpeak is a free service for non-commercial small projects (<3 million messages/year or ∼ 8200 messages/day). Four annual license types are offered for larger projects or commercial applications.

ThingSpeak provides a cloud-based service where users can create “channels” to receive data. Each channel acts as a repository for data streams, and each stream corresponds to a specific sensor reading, such as temperature. The Ardulake temperature profiler must be associated with a Read API key and a Write API key specific to each ThingSpeak channel, ensuring secure and authorized communication.

Upon receiving data, ThingSpeak organizes it into fields within the designated channel. Each data point is time-stamped, allowing for precise tracking and historical analysis. Once data is stored in a channel, ThingSpeak provides various visualization tools. Users can create custom charts and graphs to display the data in real-time or over specific periods. These visualizations are accessible through a web interface, making monitoring the equipment’s performance and analyzing trends easy.

Those who build a new Ardulake temperature profiler must register on the ThingSpeak website, create a new channel, configure the private and public views, and generate two 16-digit API Keys (Read and Write API Keys). Copy these codes and modify the Arduino code (substitute yellow-marked text by de code, without spaces).

**Table:** 

//String datos=“GET https://api.thingspeak.com/update?api_key = Read API Keys&field1 = 0″ + String(temperatura0) + ”&field2 = 0″ + String(temperatura1) + “&field3 = 0″ + String(temperatura2) + ”&field4 = 0″ + String(temperatura3) + “&field5 = 0″ + String(temperatura4) + ”&field6 = 0″ + String(temperatura5) + “&field7 = 0″ + String(v2);
String datos=“GET https://api.thingspeak.com/update?api_key = Write API Key&field1 = 0″ + String(temperatura0) + ”&field2 = 0″ + String(temperatura1) + “&field3 = 0″ + String(temperatura2) + ”&field4 = 0″ + String(temperatura3) + “&field5 = 0″ + String(temperatura4) + ”&field6 = 0″ + String(temperatura5) + “&field7 = 0″ + String(v2);

Information about the creation of a new channel can be found in the associated repository [23].

The system will assign a numeric Channel ID. The created channel must be configured. You must edit a name and a description and include information about each variable (fields), geographic coordinates, and other optional information. The channel creator can select how the data is shown to public users. One of the menus enables API Key generation.

### 6.3 Buoy

**Fig. 2:**
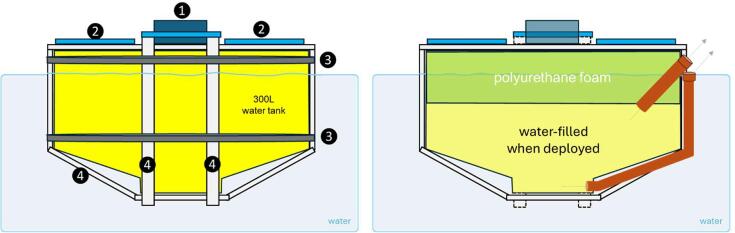
Ardulake buoy design, side view. Left, external view: 1) Rugged plastic waterproof box, 2) solar panels, 3) stainless steel straps, 4) aluminum profiles (harness). Right, internal view (cut in half, seeing inside the buoy). The brown cylinders represent pipes used to fill during deployment or to empty during withdrawal. Pictures of the design and procedures can be found in the repository.

The buoy was constructed using a triple-layer linear polyethylene water tank resistant to UV radiation. The selected tank has a cylindrical shape with a diameter of 102 cm and a truncated cone developed from the upper plane face (Fig. 2). The total height is 56 cm, and the volume is 300 L. Approximately 15 cm of the tank bottom was filled with expanding polyurethane foam, and the buoy was installed upside down. A special yellow paint for plastic surfaces was used. As the addition of solar panels, battery, and other components caused an upward displacement of the structure’s center of gravity, buoyancy and anti-roll stability were iteratively adjusted in a 30.000 L pool. The final design consists of filling the open space of the tank with approximately 180 L of lake water. No additional deadweights were needed in the final prototype. Water pipes were installed to enable the flow of water during the deployment or retrieval process (Fig. 2). Water transfers were made using a portable motor pump.

Structural reinforcement was achieved using two pairs of vertically crossed surrounding structures. Two parallel vertical rings were created using rectangular profile aluminum tubes reinforced with angles of the same material, pop rivets, and stainless-steel screws. An equivalent structure of two vertical rings was installed perpendicularly. This aluminum harness allows the installation of a rugged plastic waterproof box in the top center of the buoy and four solar panels aligned with each lateral face of the box. Weather-resistant plastic straps were used to join the box and panels to the metal structure. Cable glands and silicone ensure the tightness of cable routing. Humidity was controlled using containers with silica gel, which were periodically replaced.

Two horizontal rings were installed using stainless steel straps and buckles to provide extra strength and structural unity. Finally, a ring of nautical quality rubber-coated stainless steel rope going through aluminum perforations was used with cable clamps as fixation points for nautical ropes. Full sun and no weather shelter impose the use of high-quality materials. Galvanized components were tested and discarded, even for freshwater conditions.

## 7 Operation instructions

**Fig. 3:**
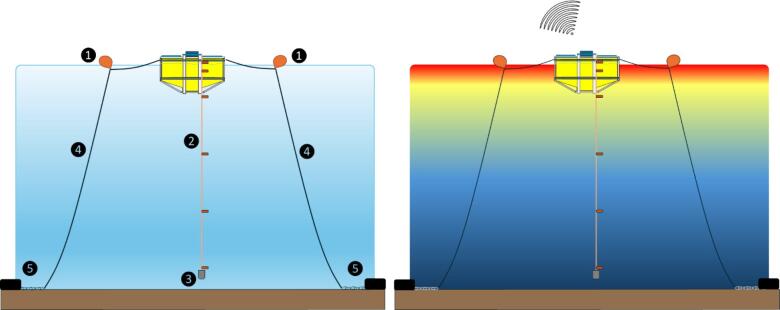
Proposed deployment layout for the Ardulake system. Left: 1) accessory nautical buoys, 2) string of temperature sensors, 3) 300-gram ballast, 4) nautical ropes (Ф 10–12 mm), 5) mooring chains and deadweights. Right: representation of vertical temperature gradient and data sending.

The system was installed at the lake’s deepest point (see validation and characterization). A series of considerations were taken to maintain the horizontal buoy position and prevent breaking the lines associated with water level variation. From that, two small accessory nautical buoys (Fig. 3) and chains were used to tighten the ropes from above and below correspondingly. Two concrete deadweights were positioned linearly on opposite sides of the buoy for mooring.

The system requires low maintenance. Data telemetry lets us notice battery problems, detect improper data behavior, and act reactively. Battery life was around two years.

Preventive maintenance mainly involves assessing box water leaks, replenishing dry silica gel, and cleaning solar panels. During maintenance, we turn off the equipment to avoid unintended interference with real-time operations. These practices have assured the system’s integrity and eliminated data collection without environmental meaning caused by human interaction.

**Fig. 4:**
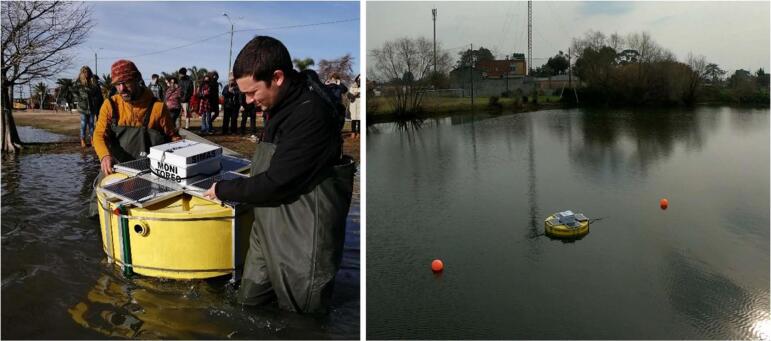
Pictures of deployment and installed monitoring buoy on Shangrila’s Lake.

After installation, the three buoys (the monitoring one and the two complementary ones) should remain linear (Fig. 4). Any deviation from this linear arrangement establishes an alert of drift of mooring components or breakage of nautical ropes that must be promptly fixed. Multirotor drones are a very useful tool for visual inspection from the shore. A laptop and a USB cable are needed to connect with the Arduino Nano, but only to upload code after becoming corrupt.

There are no inherent risks associated with the use of the equipment. However, all relevant precautions must be considered since it is a piece of field equipment, and its installation and access involve navigation. Last, when birds use the buoys, safety measures should be taken to maintain safe working conditions for operators against avian influenza or other zoonoses.

We include a document containing a visual archive showcasing the development of the Ardulake temperature profiler in the associated repository [23]. The selected images chronologically document the evolution of the Ardulake system, from the prototype version 1.0 (2019), developed with the participation of high school students, to later iterations that included more advanced configurations and components such as solar panels, sensor arrays, and buoy preparations. The archive highlights key milestones such as the deployment, maintenance, and design adjustments made to the system in 2021.

## 8 Validation and characterization

The monitoring capacity of the Ardulake temperature profiler was tested from July 2020 in Shangrilá’ Lake (−34.8523, −55.9927), a public ecosystem under the vigilant oversight of a community dedicated to environmental stewardship. This system is one of several artificial lakes formed due to sand extraction in the second half of the 20th century [23], and currently is part of the landscape of Ciudad de la Costa. As the lakes age, their internal load of nutrients increases, leading to eutrophication, a widespread environmental problem [23].

**Fig. 5:**
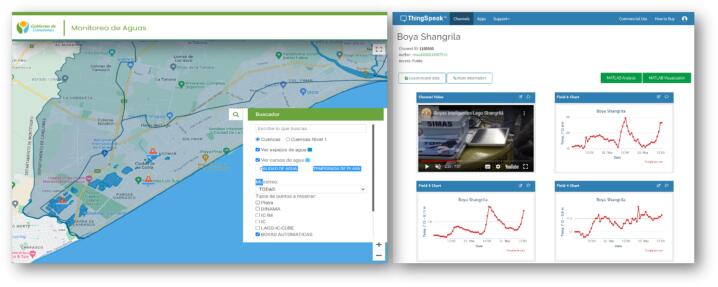
Raw data is accessed through the institutional website of Intendencia de Canelones [24] and the Thingspeak data interface (left).

The Shangrilá’ Lake is 5 m in maximum depth, and its environmental condition depends on the time of year and weather. The level of understanding of the links between ecosystem structure, functioning, and environmental quality is fragmented and preliminary, but a large monitoring and research effort is underway [23]. Generated data is freely accessible through the thinkgspeak.com site and the website and mobile app of the integrated water and beaches monitoring system of Canelones (Fig. 5). The Thinkspeak platform can be used without charge for non-commercial projects. For the one year beginning 6/21/2020, 95.9 % of the data that should have been acquired were recovered. The data loss was distributed sporadically, with GPRS network connectivity issues unrelated to the Ardulake System being the most probable cause.

**Fig. 6:**
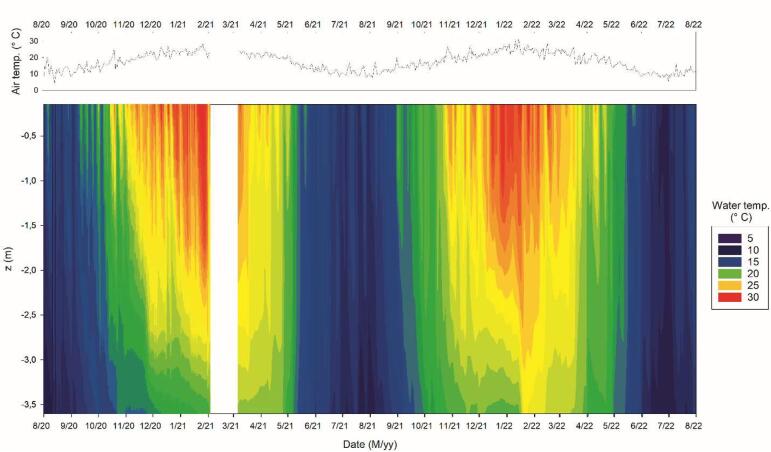
Graphical representation of generated data for two years since installation. Daily mean air temperature (above) and temperature by water depths (below). Observe spatiotemporal differences in water temperature (color contours), which resemble a warm monomictic behavior (although further research is needed). The white vertical bar corresponds to the period in which the equipment was offline by an aquatic rodent attack.

**Fig. 7:**
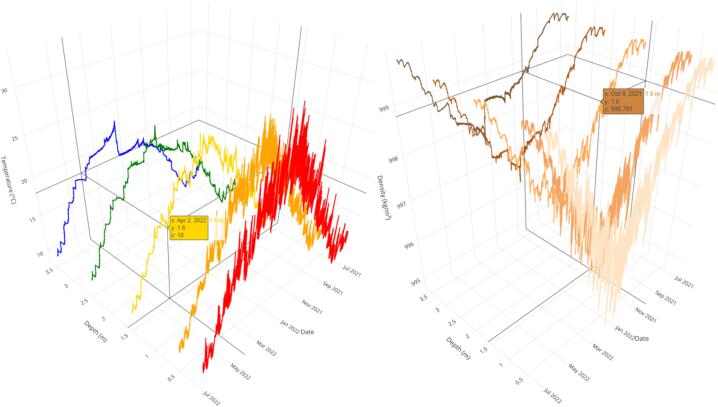
Water temperature for each monitored depth for one year from 21st June 2021 (left). Water density variability for the same period (right). The data and R scripts are available in the associated repository [23].

The monitoring buoy generates large amounts of data, which allows for detailed tracking of the thermal behavior of water mass (Fig. 6). We classify each instantaneous vertical profile according to the magnitude of its thermal gradient between 0.15 and 3.6 m. For that, we applied the threshold criteria of 1° C. m^−1^, which informs about water column stability [23]. We also estimate the water density using the empirical formula proposed by McCutcheon *et al*. [25] (Fig. 7). The generated data aligned with physical expectations, revealing inverse relationships between temperature and depth, and direct relationships between density and depth.

**Fig. 8:**
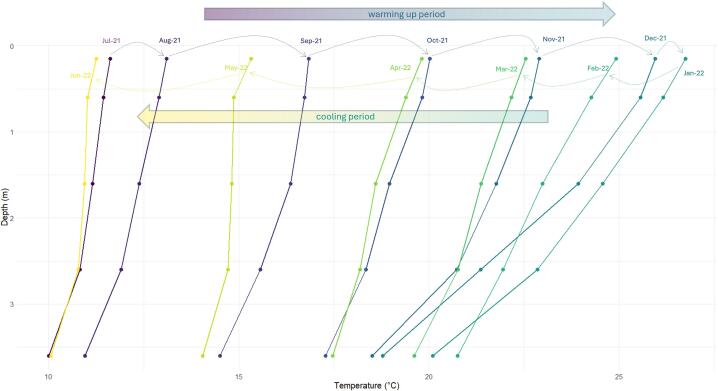
Monthly average vertical temperature profiles on Lake Shangrila (July 2021-June 2022).

A complete data set generated with Ardulake in the Shangrila’s Lake for one year from 21st June 2021 was shared on the associated repository [23]. We also estimated density, vertical temperature and density differences, and temperature gradient by meter (° C. m^−1^) considering shallowest and deepest monitored points. Shared data also includes the assigned category according to the magnitude of the vertical thermal gradient. In addition to the dataset, we included R scripts for re-generating and exploring the 3D graphics contained in this manuscript (Fig. 7). This tool allows a dynamic exploration of data, allowing rotation, changing the perspective, and zooming at the desired level. We also estimated the monthly average temperature at each water depth, which allows us to follow the temporary change of vertical profiles during warm-up and cooling periods (Fig. 8).

The high spatiotemporal detail of the available data has allowed us to advance our investigation of stratification and mixing patterns at different time scales, to establish the depth of the thermocline, and calculate parameters such as Lake and Wedderburn Numbers, Schmidt Stability, vertical seiche period, and Brunt-Väisälä buoyancy frequency using specifically developed software for high-resolution monitoring buoys [R Lake Analyzer; [26]]. Generated data is an adequate input for hydronumeric modeling [27], [28]. The research group is currently working on all these topics.

### 8.1 Main interferences

**Fig. 9:**
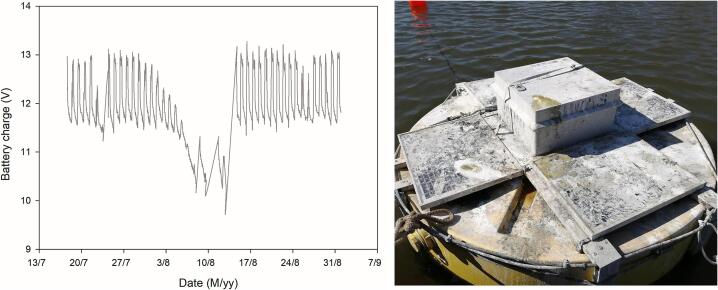
Voltage drops caused by cormorant feces (left) only a few days after deployment. Note the extreme level of shading achieved on the solar panels (right).

The learning curve after installation was mainly centered on unexpected interferences caused by biota. The first problem was detected as an abrupt voltage drop that compromised the functioning of the equipment, but without data loss (Fig. 9). The cause of these events was the use of the floating structure as a resting point by *Phalacrocorax brasilianus* (Gmelin, 1789). This species of neotropical cormorant (locally named Biguá) is a skilled swimmer that spends extended periods drying their feathering in safe spots near water bodies where they feed. To reduce their weight and facilitate takeoff, they often defecate before leaving their perch, expelling streams of liquid guano capable of staining large areas. The repetition of this habit covers the entire structure in just a few days, resulting in an intense shading effect that limits energy harvesting by the solar panels. It was necessary to design and install an aluminum pyramidal structure with a plastic net to restrict the use of the structure and reduce maintenance efforts.

The second biological-derived event configured a major issue and caused one month of lost data. Telemetry allows us to detect in real-time how the temperature data was lost during the afternoon of February 2, 2021. The cause of this loss of function was also established using a drone. A *nutria criolla* (*Myocastor coypus*; Molina, 1872), a big rodent that can reach 10 kg, cut the plastic net and gnawed all the plastic objects, including data cables. The video of the nutria on the monitoring buoy can be seen at https://youtu.be/70pEjnqiS-Y. The buoy had to be removed, and the equipment was re-assembled. After that, deterrent bird spikes were included. Other issues were related to cutting a nautical rope by strong winds but did not affect the function or data sending. We don’t find development of biofouling on the sensors.

The equipment is inaccessible to people, and no acts of vandalism have occurred. However, close work is being done to disseminate the experience, which reduces the likelihood of unwanted interferences.

The buoy functioned until September 2022, when data transmission ceased because of a short circuit in the submerged sensor cables. After the substitution of sensors, the equipment was re-installed.

### 8.2 Considerations for future developments

An increase in the number of temperature sensors is theoretically possible. Nevertheless, the maximum number of DS18B20 sensors that can be connected to an Arduino Nano via the OneWire protocol depends on several factors, such as the board’s available memory and processing power and the length and quality of the wires used. In general, we recommend to keep the number of sensors below 10 for optimal performance. However, with proper wiring and code optimization, connecting up to 20 sensors to an Arduino Nano may be possible. If you add more temperature sensors, you must customize your Arduino Nano code (please see step-by-step instructions included in the Supplementary material 3 available in associated repository [23]). It is important to note that adding more sensors may increase the risk of communication errors and may require additional power supply considerations.

Temperature sensors at greater depths probably depend on achieving sufficient protection measures to prevent dead shorts. Resin encapsulation might be the way to explore. Tests regarding resistance to freezing temperatures have not been carried out, which would be relevant for use in dimictic lakes and cold climates. From 2024, waterproof sensors with wires 10 m long are available on Amazon.com (Amazon Standard Identification Number / ASIN: B0C1L7RPJQ). We have begun testing these sensors, as their longer cables eliminate the need to extend the 3-meter cables by joining submerged contacts.

One of the weaknesses of the design is the monitoring system’s limited data storage capacity. If the data isn't transmitted after each reading (due to low cellular signal), it will be lost. We are actively working to address this issue. On the other hand, one of the strengths of the system lies in its low construction costs, which make it possible to create backup units that can be quickly deployed in case of problems, thereby minimizing data loss.

The GSM module SIM800L supports only 2G services, which can be a problem to fix next. In our experience, only one of the three local phone companies has decided to keep the 2G signal. We are now working on testing an LTE module. Other development perspectives include miniaturization, which depends largely on more accurate estimates of electrical consumption. Reducing the size of the buoy depends mainly on the use of fewer solar panels, which in turn reduces costs and potential unwanted interactions with animals.

## Ethical approval

This study does not present any ethical considerations relevant to the guidelines of HardwareX.

## CRediT authorship contribution statement

Guillermo Goyenola: conceptualization, funding acquisition, project administration, investigation, infrastructure building, fieldwork, supervision, data curation, formal analysis, original draft writing, review & editing. Javier García-Climent: electronic design and programming, adaptations and improvement of equipment, infrastructure building, fieldwork, review & editing.

## Declaration of competing interest

The authors declare that they have no known competing financial interests or personal relationships that could have appeared to influence the work reported in this paper.
